# Benefits and barriers associated with the use of smart home health technologies in the care of older persons: a systematic review

**DOI:** 10.1186/s12877-024-04702-1

**Published:** 2024-02-14

**Authors:** Yi Jiao (Angelina) Tian, Nadine Andrea Felber, Félix Pageau, Delphine Roulet Schwab, Tenzin Wangmo

**Affiliations:** 1 Institute for Biomedical Ethics, University of Basel, Basel, 4056 Switzerland; 2 Centre d’excellence sur le vieillissement de Québec, VITAM– Research Center on Sustainable Health, Laval University, Quebec City, QC Canada; 3 Division of Geriatrics, Department of Medicine, Laval University, Quebec City, QC Canada; 4 School of nursing sciences, La Source, HES-SO University of Applied Sciences and Arts of Western Switzerland, Lausanne, Switzerland

**Keywords:** Older people, Independent living, Smart home health technologies, Caregiving, Home

## Abstract

**Background:**

Smart home health technologies (SHHTs) have been discussed in the frame of caregiving to enable aging-in-place and independence. A systematic review was conducted in accordance with the PRISMA guidelines to gather the up-to-date knowledge on the benefits and barriers of using SHHTs in the care of older persons from the perspective of older persons and their caregivers.

**Methods:**

Ten electronic databases were reviewed for empirical peer-reviewed literature published from 01.01.2000 to 31.12.2021 in English, German, and French reporting on experimental, qualitative, quantitative, and other empirical study designs were included. Included studies contained user-feedback from older persons over 65 years of age or their caregivers (formal and informal). We used an extraction document to collect relevant data from all included studies and applied narrative synthesis to analyze data related to benefits and barriers of SHHTs.

**Results:**

163 empirical peer-reviewed articles were included, the majority of those published between 2014 and 2021. Five first-order categories of benefits and five of barriers were found with individual sub-themes. SHHTs could be useful in the care context where continuous monitoring is needed. They improve self-management and independent living of older persons. Barriers currently exist with respect to ease of usability, social acceptance, and cost.

**Conclusions:**

SHHTs could be useful in the care context but are not without concerns. Researchers and policy makers can use the information as a starting point to better understand how the roles and outcomes of SHHTs could be improved for the care of older persons, while caregivers of older adults could use our findings to comprehend the scope of SHHTs and to decide when and where such technology could best address their individual family needs. Limitations lie in the possible exclusion of relevant articles published outside the inclusion criteria as well as the fact that due to digital divide, our review represents opinions of those who could and wanted to participate in the included 163 studies.

**Trial registration:**

This review has been registered as PROSPERO CRD42021248543. A protocol was completed in March 2021 with the PRISMA-P guidance. We have extended the review period from 2000 to 2020 since the registration of the protocol to 2000–2021.

**Supplementary Information:**

The online version contains supplementary material available at 10.1186/s12877-024-04702-1.

## Introduction

### Rationale

Recent developments in medicine, public health, and medical technologies have led to an increase in life expectancy and an upwards trend in the global aging population [1]. At the same time, these trends are coupled with the rising likelihood for older adults to have increased risks of frailty, falls, disease and a reduced or loss of independence in completing instrumental activities of daily living (IADLs) (running errands, managing finances, using a computer and phone, etc.), and other ADLs (bathing, getting dressed, feeding oneself, etc.) [2]. To support and manage these declining abilities to independently undertake IADLs and ADLs, both informal and formal caregivers must provide extensive care and supervision, or alternatively consider a move towards institutionalization. Nevertheless, previous studies indicate that most older persons express negative feelings towards uprooting their lives from their homes, which may call for a solution that allows older persons to age-in-place while illness-appropriate and timely care could also be provided [3, 4].

With recent technological advances in the field of connected devices and the Internet-of Things (IoTs), homes could be rendered “smart” by being fitted with unobtrusive, non-invasive, and wearable or stand-alone assistive health devices that communicate with each other, other systems, and end-users [5, 6]. The definition of the “smart home” used in this paper is from Demiris and Hensel (2008), which is: “a residence wired with technology features that monitor the well-being and activities of their residents to improve overall quality of life, increase independence and prevent emergencies” [7]. To take this definition into more modern contexts with current advancements in wireless computing and application, this paper focuses on the empirical studies investigating all technologies available from 2000 to 2021 for health-related care and support in a home or residence context. These smart home health technologies (SHHTs) are categorized into 6 types: (a) physiological monitoring, pertaining to the collection and analysis of physiological measurements like heart rate, blood oxygen levels, blood pressure, respiration, temperature, or weight, etc.; (b) functional monitoring of data, including movements and activity levels while walking, sleeping, and eating, along with detecting abnormal movements or postures; (c) safety monitoring and assistance in the home environment for wandering behaviors, reduction of risks of falls or trips through automatic light switches in bathrooms during nighttime etc.; (d) security monitoring and assistance for the detection and responses towards intruders or threats; (e) social interaction monitoring and assistance with additional communication channels for health and well-being information or virtual participation in social events; and (f) cognitive and sensory assistance, including reminder systems for medication or cognitive aid functions for locating objects or practical instructions to aid forgetfulness [7]. The aspects of interoperability and automation in smart home technologies allow various different devices fulfilling the array of these health-related functions to communicate with one another [5, 8, 9]. Therefore, each health-related device in a residence is not a stand-alone entity but is compatible to be controlled and configured as connected ecosystem of technologies. For example, rather than measuring blood pressure, sleep time, and falls using individual devices at home, then operating and inputting the results onto another interface for a health-visit, the remote and continuous health monitoring function made possible with the installation of interoperable sensors enable any abnormalities in the older persons’ daily habits, postures, meals, and vital signs to be congregated and reported in real-time via another user interface or voice assistants to formal and informal caregivers, where they could continue to provide remote support while aging-in-place.

On a macro level, the concept of “smart homes” has been received positively and its global markets are predicted to grow [10]. Nevertheless, we agree with Wilson et al [11] that without evaluation and adoption by their actual end-users into the context of normal lives, their overall effectiveness as a solution for caregiving purposes would stay as theoretical potentials and assumed benefits. Most of the literature reviews and research are “pushed” by technology developers and still lack the feedback of end-users [11, 12]. More research is needed to empirically investigate SHHTs from the end-users’ perspective, drawing in the issue of acceptance and adoption in the context of the personal environment that these technologies are used in [13].

Different from previous reviews of literature on smart home technology and caregiving, we narrowed the focus from reviewing all smart home devices similar to the work of Wilson, Hargreaves and Hauxwell-Baldwin [11] to SHHTs by adding the limit of the use of health technologies. We believe that this focus on health technologies, rather than those geared towards comfort, convenience, and entertainment could more directly address a few of the major concerns in the aging process, such as frailty, cognitive impairment, age-related disabilities, and risks of mortality [14]. The systematic review by Majumder and colleagues [5] focused on specific types of SHHTs used for older persons, such as wearable sensors, or another recent review by Pirzada and colleagues [15] that did not include assistive robots, whereas our more comprehensive review allows an aggregate overview of devices that fulfill a more diverse portfolio of interactive health needs and personal preferences. Namely, the installation of home sensors also involves the monitoring of health and signs of diseases, but could resolve the issue of forgetfulness common in the adoption of wearable sensors, concerns of waterproofing when the older person is in the shower, or the preferences against wearing devices on the body. The companion robots could address the angle of social isolation, while service robots could allow us to look futuristically towards the new features for hands-on, rather than monitoring functions that technologies could provide. Also different from those that focused on a specific topic in relation to SHHTs, such as loneliness and social isolation from Latikka et al. [16] and Choi and Lee [17], our systematic review provides an overview of benefits and barriers while presenting individual issues within a broader perspective of well-being, health, and an improvement of the quality of life of both the older persons and their caregivers. Therefore, we not only heed the calls from existing reviews to empirically examine from an end-user’s perspective, this review also differs from and adds to the works of our colleagues by focusing not only on, but all those, SHHTs used for caregiving purposes with sampling of older persons or those directly involved in their care.

### Objectives

Our systematic review aims to capture the existing knowledge including barriers and opportunities in the uptake of SHHTs in the care of older persons. Specific research questions include (1) What are the benefits and opportunities that SHHTs bring to the caregiving context in the existing empirical literature? (2) What are the barriers to acceptance or areas of improvement in SHHTs when they are used to care for older persons in the existing empirical literature?

## Methods

### Search Strategy

**Fig. 1:**
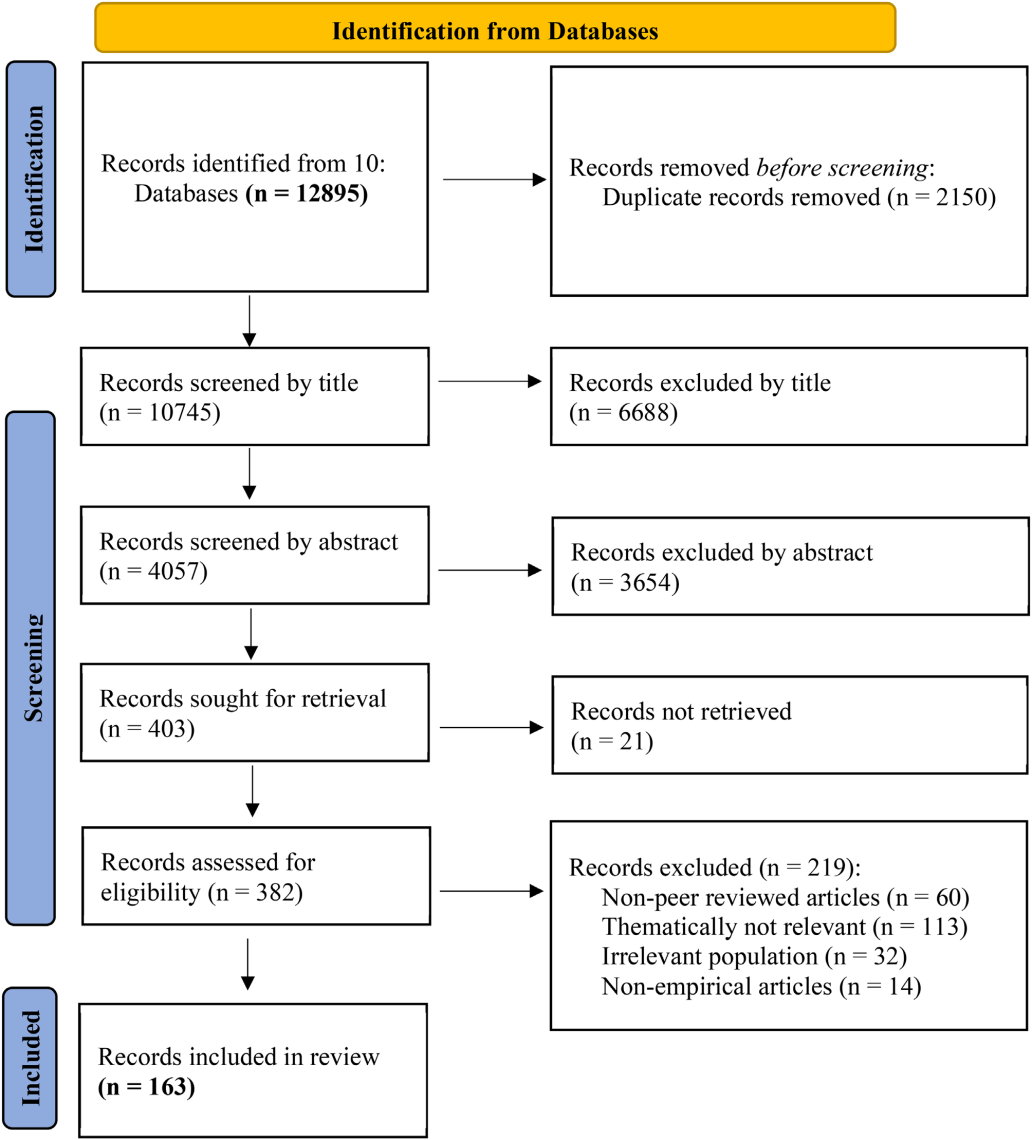
PRISMA 2020 flowchart

To capture the relevant intersection between older persons, caregivers, and SHHTs, we used a search algorithm (see Table 1) organized into four PICO (Population, Intervention, Context, Outcome) categories covering facets of interest: Population 1 (Older adults), Population 2 (Caregivers), Intervention (Smart home technologies), and Context (Home). By “home”, we mean an individual’s place of residence. This would include not only one’s home or apartment in the case of those older persons living in the community but also establishments providing residence and care such as retirement homes, nursing homes, aged care facilities, and assisted living that allows some level of independence for older persons. Each category included synonyms and varying spelling of each term, while also accommodating for possible definition and structure variabilities. The search strategy was also developed by the research team with the help of an information specialist. This algorithm was then adapted to use in ten digital databases: EMBASE, Medline, PsycINFO, CINAHL, SocIndex, SCOPUS, IEEE, Web of Science, Philpapers, and Philosophers Index (See Fig. 1). All terms were coupled with database-specific thesaurus terms where available. The search was limited to English, French, and German peer-reviewed papers published between 1 January 2000 and 31 December 2021. This date range was chosen to obtain a comprehensive review of existing studies, while taking into account the time of emergence and development trajectory of SHHTs, such as the advancement of wired to wireless devices or the increase in the number of interoperable multi-functional devices in the home. Although some reviews may purposively forgo searching for publications prior to 2010 (i.e. Liu, Stroulia [3]’s studied SHHTs’ technological readiness and their evidence to support older adults at home between 2010 and 2014), we believe that as the definitions and empirical work pertinent to early developments of smart homes began emerging as early as 2003 and all throughout the 2000s, there was ample need to include possible research outputs during these years [7, 18, 19].

**Table 1:** Key search terms and search strategy*

PPCC	Search term	Synonyms
Population 1	Older adults	“older adult*” OR “ag*ng” OR “elder*” OR “senior*” OR “geriatr*” OR “gerontolo*” OR “aged person” OR “older person*” OR “elderly people” OR “older parent*”
Population 2	Caregivers	“caregiv*” OR “informal caregiv*” OR “unpaid caregiv*” OR “famil* caregiv*” OR “care*” OR “formal caregiv*” OR “professional caregiv*” OR “nurse*” OR “nurse-aid*” OR “physician*” OR “doctor*” OR “spouse*” OR “adult child*” OR “daughter*” OR “wife” OR “husband” OR “son” OR “relative”
Intervention	Smart home technology terminologies	“smart house” OR “welfare technology” OR “smart home” OR “smart technolog*” OR “smart living” OR “home automation” OR “wireless home automation system*” OR “intelligent living” OR “intelligent building” OR “domotic*” OR “assistive domotic*” OR “embedded health system*” OR “ehealth” OR “health monitoring” OR “home-based health technology” OR “gerontechnology” OR “gerotechnology” OR “sensors” OR “wearable*” OR “Robotic” OR “Artificial Intelligence in Eldercare” OR “Digital monitor*” OR “smart technologies to support healthy aging” OR “information technolog* for assisted living at home” OR “home-based assistive technolog*” OR “Ambient Assistive Living” OR “Intelligent Assistive Technolog*” OR “Intelligent Assistive Device*” OR “Intelligent Assistive Application”
Context	Home “home” setting as the individual’s place of residence	“Home care” or “Nursing Home*” or “Independent Living” OR “Home*” OR “house*” OR “homes for the aged” OR “assisted living facilit*” OR “retirement home*”

* The search terms have been adapted to the relevant database standards. Within PPIC synonyms are linked by Boolean ORs, between PPIC are linked with AND. Other sources: Citation tracking, reading references

### Eligibility criteria

The inclusion criteria were: (1) The included study must be empirical and peer-reviewed. That is, an article was only included if it gathered the opinions of relevant end-users, such as caregivers and older persons, on the use of SHHTs in caregiving for older persons. (2) The studied population includes older persons over 65 years of age requiring care and support at their place of dwelling (home or nursing home) and/or professional and /or informal caregivers who provide care to older persons. Informal caregivers refer to family members or friends who provide support without monetary benefits. Professional or formal caregivers are those who are paid to help older persons receive medical treatment or perform tasks in their homes. (3) The empirical study concerns the use of SHHTs in the older persons’ place of dwelling. Specifically, these smart home technologies should be integrated into the older person’s place of dwelling, providing an interoperable system of devices that serves caregiving purposes. For example, studies were included if the health monitoring technologies such as cameras, motion detectors, or wearable sensors etc., mentioned were capable of interacting with each other and automatically alert end-users via either smartphones, tablets, or alarm services operated by formal caregivers.

We excluded studies that were (1) non-empirical and /or those published as book chapters, conference proceedings, newspaper articles, commentary, dissertations, and theses. Also excluded were systematic reviews. (2) Studies that did not report on the views of our population of interest or only included the views of researchers; and (3) technologies that are used for caregiving but is not interoperable or communicable with each other over an integration platform, such as stand-alone health devices such as those measuring blood pressure or weight, or the use of the video calling technologies to enable telehealth visits. Technologies that did not fulfill caregiving purposes for the promotion of health, such as single-purpose devices for cooking, cleaning, and comfort, were also excluded.

### Selection process

After conducting a systematic search with the algorithm (see Table 1), results across all ten databases were consolidated and uploaded to a referencing software, where duplicates were removed automatically. From this point, all screening processes for format, content, and exclusion of additional duplicates were done manually. All titles were first screened manually by the third author. Thereafter, the abstracts of remaining articles were assessed independently by the first and third authors. Disagreements and uncertainties were resolved by the second author, who also proceeded to combine all included articles from the first and third authors and removed any duplicates generated during the abstract screening.

### Data collection process

Upon screening both titles and abstracts, full electronically-available copies of remaining articles were retrieved and carefully studied by the first, second, and last authors for data extraction. At this stage, many papers were further deemed ineligible and were excluded with documented reasons. In order to identify appropriate data for extraction, the team developed a customized extraction document detailing information relevant to study demographics, technology specificities, benefits, and barriers, which was tested and adapted using several publications fulfilling the inclusion criteria. For relevant data to be extracted, it was not necessary for the article to use the exact wording of a theme already present on the extraction document. For example, though we were interested broadly in the concerns for the user-friendliness of a device, the researcher would extract data that also mentioned terms such as “slow, required directions, or anxious for making mistakes” which were not limited to whether the device was explicitly evaluated as “easy or difficult to use.” Nevertheless, articles that evaluated user-friendliness or design of a device without the collection of end-user opinions and experiences of a SHHT was not sufficient for extraction.

### Ensuring quality of collected data

All data was extracted and coded first using the extraction document by the first, second, or last author, who were each responsible for a portion of articles. During the data extraction process, they discussed any challenges that they were facing in data interpretation and how to consolidate differences, where existed. To avoid bias between the primary coders, two external researchers (the third and fourth authors with backgrounds in geriatrics and psychology) additionally analyzed 10% of all articles, and achieved 80% in consistency in content. For the quality assessment, we used the Critical Appraisal Skills Programme (CASP) Checklists (e.g. CASP qualitative research, CASP case control, CASP cohort studies).

### Data synthesis

The final extraction documents were combined across the three primary coders and data were analyzed first according to benefits or barriers to uptake of the SHHTs. Data synthesis was carried out by the first and last authors using narrative synthesis as it is most suited to combine different research designs and thus comprehensively inform policy [20]. During the data synthesis process, the two authors decided to reorganize similar columns and present them as sub-themes within several first-order categories by virtue of preserving precision while increasing comprehensiveness. Though the data extraction document with pre-existing themes was prepared in advance, the organization of final themes was dynamic, where we continuously discussed among authors on the best way to understand and subsequently portray the data as objectively and comprehensively as possible. A table containing basic information for all included articles can be found in the supplementary files.

### Protocol

Prior to implementing the search, the authors completed a protocol in accordance with PRISMA-P and registered the systematic review on PROSPERO (CRD42021248543) [21]. The review is designed and implemented in accordance with the Preferred Reporting Items for Systematic Reviews and Meta-Analyses (PRISMA) statement [22, 23]. We have extended the review period from 2000 to 2020 since the registration of the protocol to 2000–2021.

## Results

### Study selection

The search algorithm revealed 12,895 articles across ten databases for the 22 years of study time period. After removing duplicates and independently reviewing titles and abstracts, 403 articles were included at this stage. We sought to retrieve the full-text of all these articles and 21 full-texts were not found. Hence, 382 full-texts were further assessed for eligibility and data extracted where appropriate. During this process, 219 articles were excluded with reasons (see Fig. 1). This systematic review finally included 163 empirical articles for data analysis and the results are reported below.

To meet our eligibility criteria, many articles that fulfill some but not all of the required facets were not included in the final review. For example, a study on sleep disturbance in persons with dementia by Harris and Grando [24] that tested non-interoperable devices such as actigraphs as the sole technology component was also excluded, albeit on nursing home residents over 65 years old and its overall relevance for caregiving.

### Study characteristics

Of the 163 articles included for this review, 117 articles (72%) were published between 2014 and 2021, 37 (23%) between 2007 and 2013, and only 9 articles (5%) between 2000 and 2006. Almost all, with the exception of 3 articles, were published in English. The included studies encompassed a variety of empirical methodologies (see supplementary file). Furthermore, we also categorized the articles by functions of SHHTs tested or studied (Table 2), whereby the most studied function included physiological and functioning monitoring technologies (30 articles, 18.40%) followed by those solely promoting social interactions (28 articles, 17.18%). A significant portion of the locations of the included studies were in the U.S. (34 articles, 20.86%), with others in Japan, U.K., Germany, and Canada (Table 3).

**Table 2:** Function of studied technology

Studied Technology	Number of articles	Percentage of all papers
a) Physiological and functional monitoring	30	18.40
b) Social interactions	28	17.18
c) Safety/security monitoring and assistance	16	9.82
d) Cognitive and sensory assistance	9	5.52
e) Combination of above	80	42.94
	***Combinations****N*>*** = 80***	
	*a) c)*	33 (20.24)
	*a), b), c), d)*	14 (8.59)
	*b) d)*	11 (6.7)
	*a), c,) d)*	6 (3.7)
	*a), b), c)*	5 (3.1)
	*a) b)*	4 (2.4)
	*a), b), d)*	2 (1.2)
	*c) d)*	2 (1.2)
	*b) c)*	2 (1.2)
	*b), c), d)*	1 (0.6)
	*a) d)*	0 (0.0)

**Table 3:** Study Locations

Study Location (Regions)	Number of articles	Percentage
North America (USA and Canada)	41	25.15
E.U.*	41	25.15
East and Southeast Asia**	20	12.27
Combination of European Countries (non-specified)	17	10.43
Oceania (Australia and New Zealand)	12	7.36
EEA (Switzerland and Norway)	10	6.13
U.K.	10	6.13
Middle East***	5	3.07
Multi-regional****	5	3.07
Not reported	2	1.23

Locations included in above regions in order of frequency:

*E.U.: (Germany, Finland, France, Spain, The Netherlands, Sweden, Italy, Ireland, Denmark, Belgium, Greece, Austria)

**East and Southeast Asia: (Japan, Singapore, Taiwan, Hong Kong PRC, South Korea)

***Middle East (Turkey, Israel, Saudi Arabia)

****Multi-regional: (South Korea and US; France and Taiwan; Japan. Finland, Ireland; The Netherlands; US, Denmark and Japan)

### Types of technology reviewed

Often the articles also involved technologies that fulfilled one or more of the functions noted in Table 2. We also categorized all 163 articles into the specific types of emerging technologies (Abdi, de Witte & Hawley, 2020) and found that the two often mentioned technologies were intelligent homes (77 articles; 47.24%) and assistive autonomous robots (57 articles; 34.97%). Articles that fall under (1) intelligent homes investigated a collection of real-time monitoring sensors, such as cameras, infrared motion sensors, environmental sensors installed in the bed-mattress, contact sensors on doors, or pressure sensors detecting movements on the floors. (2) Autonomous robots include companion robots such as Pepper and Zora for entertainment and social interactions, or those combined with assistive functions like Care O Bot. In addition, we also included devices that could monitor for the frequency of social interactions of older persons and their families, as well as the inclusion of wearable devices monitoring for vital signs and activity levels within the wider package of smart home technologies under (3) AI-enabled health smart apps and wearables (5 articles; 3.07%). Due to the inclusion criteria of SHHTs needing to be home-based, we did not include articles that only investigated the uses of smart watches and fitness trackers that could be used outside of the home, namely for their GPS locating functions. (4) Voice-activated devices (3 articles; 1.84%) are those already on the market, such as Amazon Echo or Google Home, which articles are only included if they are tested on older persons for caregiving purposes. (5) Drug release mechanisms (2 articles; 1.23%) included smart pill dispensers that ejected specific numbers and types of medication in the homes of older persons, provided reminders when medications are not taken, which was all synced with interfaces accessible by caregivers.

### Benefits mentioned in the included studies

From the 163 articles reviewed, we categorized benefits associated with the use of SHHTs into five main categories (see Table 4), which are described below.

**Table 4:** Benefits of smart home health technologies in the care of older persons

Main Benefits of Technologies	Mentioned by # of articles	Sub-Themes
Continuous monitoring of the older person	115	a) Detection of abnormal postures, falls
		b) Provide information to allow better caregiving
		c) Detect declines in functioning
		d) Ensure safety
		e) Provide assurance
Social interactions of older persons	74	a) Promote relationships between caregivers and older persons
		b) Facilitate communication with others
		c) Improve mental state and emotions
		d) Engage and form social bonds
Promotes independence or independent living for older persons	67	a) Supports basic activities for daily living
		b) Reduce reliance on children
		c) Understand habits, personal routines and provides a sense of security
Reminds older persons to self-care and self-management	49	a) Medication management
		b) Knowledge of own health conditions and medical information
		c) Promotion of a healthier lifestyle
		d) Memory aids
Other opportunities and benefits	75	a) Reduce caregiving burden
		b) Improve well-being
		c) Support caregivers and healthcare systems
		d) Entertainment purposes

1. *Enables continuous monitoring of the older person*: This category is mentioned in 115 articles, which represented more than two-third of all articles. Within this category, included studies mentioned technologies that could detect abnormality in postures, movements including falls, signs of agitation, and lights turning on at abnormal times (e.g. [25–28]). Such monitoring information were deemed as relevant information about the older persons’ health and habits that could be recorded and provided to the caregiver, which makes caregiving simpler, more efficient, holistic, or of higher quality (e.g. [29–32]). Furthermore, technologies could also be used to detect declines in the older persons’ cognitive or physical functioning through data related to periodic forgetfulness, vital signs, or mental health state (e.g. [33–37]). Technologies with a monitoring function also works to ensure the older persons’ safety in the home environment, in both aspects of home security and emergency situations (e.g. [38–43]). Technologies could also give confidence and assurance to the older persons that they are watched over and their knowledge about their own habits are valid (e.g. [44–46]). At the same time, older persons are also happy that technologies could provide peace-of-mind to their caregivers (e.g. [47]). Lastly, caregivers anticipate the possible usefulness of this function by enabling the prediction of fall risks from the older persons’ performance on mobility tasks [48].
2. *Encourages social interactions*: 74 articles mentioned benefits associated with promoting relationships, social exposure, and decreasing loneliness. Technology was cited to improve relationships between older persons and their caregivers, both in terms of quality and quantity (e.g. [43, 49–52]). Specifically, technology facilitated communication with others through additional means, such as video calls, social media, or simply through a virtual interface (e.g. [53–57]). Technologies could improve older persons’ mental state by decreasing negative emotional states, such as loneliness and symptoms of apathy (e.g. [58–61])., enhancing social bonds by reminiscing about their past, and forming new social bonds (e.g. [62–65]). One article cited an older participant becoming more relaxed and positive, as well as more accepting of other people from interacting with the Paro robot [66].
3. *Promotes independence or independent living for older persons*: This category was discussed across 67 articles. Mobile assistive robots helped the older person with basic ADLs, such as walking, showering, and picking up objects from the floor (e.g. [67–70]). Such support was reported to allow older adults become less dependent on care from formal and informal caregivers, thereby making them less reliant on children and more independent (e.g. [71, 72]). Digital medication dispensers, social robots, and pervasive sensor-equipped home systems could give older persons a sense of independence, self-determination, and empowerment in the home environment by giving them confidence to function well and be in control of their own health at home instead of asking for a human caregiver to come by to attend to their every need (e.g. [73–76]).
4. *Reminds older persons to promote self-care and self-management*: This category is mentioned across 49 articles. These strategies included the ability of SHHTs to support medication management, thus requiring less support from caregivers (e.g. [75, 77–79]). Some enable older persons to analyze their health conditions and making medical information available to them (e.g. [80–82]). Others provided recommendations and feedback pertinent to diet, physical or cognitive exercises, as a way to encourage older persons to become more motivated to live healthier and happier lifestyles (e.g. [38, 83–86]). Lastly, older persons could also be provided with memory aids for tasks other than medication, such as reminders for keeping appointments, guidance for completing tasks, and the reorientation to time and place (e.g. [87–90]).
5. *Other purposes*: 75 studies included all the remaining benefits raised, which were comparatively more scattered in definition than the preceding categories. For example, studied technologies were cited to reduce caregiving burden through decreased visits, time, and money combined with increased freedom, peace of mind, and support in caregiving tasks (e.g. [66, 91–95]). Such technologies could improve older persons’ well-being, specifically in regard to memory, emotional and physical health, sleep, and communication (e.g. [57, 96–100]). More broadly, they could support caregivers and healthcare systems, improving satisfaction and confidence with work, morale, and healthcare delivery cost and quality (e.g. [101, 102]). Lastly, studies found that technologies could entertain older people with games, jokes, music, and humor (e.g. [103–105]).

### Barriers mentioned in the included articles

From the 163 articles reviewed, we categorized barriers to the use to SHHTs into five main categories (see Table 5), which are described below.

**Table 5:** Barriers to the use of smart home health technologies in the care of older persons

Main Barriers of Technologies	Mentioned by # of articles	Sub-Themes
Usability	110	a) Design was not conducive for easy use
		b) Disruptive to end-user
		c) Technical problems with battery life, connectivity, incompatibility
		d) Low technology maturity
		e) Should account for functional limitations
		f) Design should be more reliable and accurate
		g) Should be customizable
Social acceptance	69	a) Valuable for future needs
		b) Formal caregivers perceive benefit
		c) Too difficult or annoying to use
		d) Others may not approve
Cost-related issues	44	a) Not affordable
		b) Would use if financed or reimbursed
		c) Informal caregivers less sensitive to cost, but should not have a right to disrupt choices of older persons
		d) High cost could result in distributive injustice
Loss of relationships or increase of loneliness	22	a) Fear the loss of human contact
		b) While family caregivers do not believe technology could weaken their commitment
Other concerns	25	a) Increase workload of caregivers
		b) Unnecessary or useless, only a portion generated useful information
		c) Lack of clarity to data processing

1. *Usability*: In 110 studies, users thought the tested technology was not easy to use, intuitive, or conducive for use and was too bulky and ugly (e.g. [39, 53, 80, 106]). Fear of a robotic device due to the unfamiliar and humanoid appearances could also lessen the frequency of interactions of older residents [107]. Technologies’ use was found to result in anxiety or destabilization of the end-user or complaints with the alert system, wearable, and sensors (e.g. [96, 108–110]). Technical problems pertinent to battery life, internet connectivity, incompatibility with existing home systems, and password entry were also mentioned (e.g. [111–114]). Users were also unsatisfied with the technical limitations such as short screen time and long starting time or the inability to identify wrong postures, for instance, in fitness classes (e.g. [35, 78, 115, 116]). There were suggestions for more interactive feedback from the devices to guide users, such as those to indicate the completion of a task or a physiological reading. Some complained that the technology was simply too disruptive and intrusive (e.g. [87, 117, 118]). On the other hand, articles also suggested possible improvements such as accounting for the visual, auditory, language barriers, cognitive declines and other limitations and wishes of older persons (e.g. [34, 62, 101, 107, 119, 120]). For example, robotic assistants should be able to speak the language of the users, or devices requiring older participants’ engagement should be split into smaller, sporadic checks to account for cognitive declines in persons with dementia [107, 121]. Reliability should be improved with less malfunctions and more accurate alerts and warnings (e.g. [27, 69, 122, 123]. Lastly, technologies should be adaptive and customizable, that allows end-users with a variety of capabilities, needs, and preferences to maximize their benefits for caregiving [60, 120, 124].
2. *Social acceptance*: 69 articles elicited the factors affecting end-users’ decisions for technology adoption. Some end-users found the technology valuable and saw its utility in the future with increasing age and needs (e.g. [46, 83, 86, 104, 125]. In addition, the technology would be accepted when it addresses an actual need of an older person, thereby making it a necessity (e.g. [125–127]). Formal caregivers would accept the technology given its positive benefits for the workplace and the older patients (e.g. [62, 112]). A few articles also recorded hesitancy to use technologies. For example, some users believed that the tested SHHTs are too difficult for older persons to use, citing too old to learn and keep up with changing technology (e.g. [51, 114, 117, 128]). Older persons also worried that family members or the public may not approve, and the device may generate unwanted attention or gossip (e.g. [48, 62, 102, 129]. In addition, not only did the perception of family member interfere with the older persons’ preferences or uptake of technologies, the relatives of older persons also preferred to be involved in the approval or opinion-gathering processes of the planned usage of technologies [130, 131]. Lastly, technology itself could be deemed annoying due to the frequency or presence of alerts (e.g. [132].
3. *Cost/Affordability*: 44 studies questioned the cost-effectiveness and affordability of the tested technologies, noting to both their initial purchase and continued maintenance (e.g. [35, 95, 104, 113, 133])., though there was anticipatory comments that sensor usage could reduce healthcare costs and become an effective solution for caregivers and older persons [37]. However, the technology could be used if it was financed or reimbursed (e.g. [119, 134, 135]). This financial reimbursement extended beyond technologies, but their counselling services from healthcare providers, as this also affected the accessibility issue for many end-users [131]. While informal caregivers are less sensitive to the cost compared to older persons and buys the technology, it does not give them the right to disrupt the life-style choices of older persons [54, 68]. Finally, a few papers pointed out that the high cost of technology risks to create a difference between the haves and the have-nots, such as those with in rural areas with no internet connection, and hence a better option would be when technology are made available to everyone (e.g. [37, 46, 71, 92]).
4. *Loss of relationships or increase of loneliness*: Within the potential loss of relationships, two opposite issues were highlighted across 22 included studies. Firstly, there were concern and regret at the potential loss of social connectedness that older persons cannot enjoy as technology was supposed to provide for the care time that a loved one would have provided (e.g. [37, 48, 50, 71, 76, 136]. On the contrary, family caregivers did not believe that technology would weaken their commitment towards the older persons (e.g. [136]. Specifically for the use of robots, caregivers also felt that they must supervise those interactions with older persons (e.g. [99]. Moreover, there were mentions that the robot would decrease loneliness since it is waiting for the older person at home and making the home environment friendlier (e.g. [38, 72, 88].
5. O*ther concerns*: The last category cites all other remaining concerns from 25 articles. For example, there were worries especially among formal caregivers that introducing technology would increase workload by requiring ample training and time before their introduction to older persons (e.g. [62, 116, 117, 125]). This was echoed in an outlier set of articles that cited concerns with workload increases from translating conversations when the robotic agents did not speak the language of the older persons, as well as the necessity to process and react to the alerts generated by the monitoring devices [37, 86, 107]. There were also general complaints that the device is unnecessary, some of the components generated useful information, and procedural uncertainty with the generated data (e.g. [34, 132, 137]).

## Discussion

Considering the context of population aging and resource preparedness needed to support aging at home, it is necessary to consider the scope of viable solutions that could address caregiving to older persons. As a way to support caregivers by continuously providing information and monitoring abnormal health statuses of older persons, technological solutions such as SHHTs have been brought forth to improve well-being for older persons while reducing caregiving burdens. Our systematic review unearths important existing knowledge on SHHTs by studying their benefits and barriers in the care for older persons across the 163 empirical peer-reviewed publications. To supplement this review on the practical benefits and barriers to SHHTs’ adoption, we have published another article on the ethical concerns in the use of SHHTs from empirical and theoretical articles, where the issues of informational privacy in terms of data protection and security, and the impact on autonomy from the gain or loss in independence and control, as well as stigma and responsibility have been elaborated in further detail [138].

In regard to the existing benefits, most articles cited the capacity of SHHTs to allow continuous monitoring of the older person. This is not surprising as it is the one of the central challenges that many SHHTs aim to enable independently at home [10, 11, 139]. Importantly, monitoring technologies could relieve caregivers of the need to be constantly present to, for example, ensure safety by being able to find older persons who have wandered off [140, 141]. As devices within the household communicate with each other via the concept of IoT, they not only allow continuous control and monitoring, but also regulation of the residents’ movement, routines as well as habits, and of the home itself [10, 18]. As a critical extended function, older adults could choose to customize the SHHTs to alert any desired party if there are deviations from normal routines and postures. Stated in the results, end-users appreciated the ability of data from sensors to alert caregivers about any declines in cognitive or physical functioning. Having such knowledge could help in planning for increasing future caregiving needs. Furthermore, the function of medical alerts not only enables the provision of relevant emergency information to caregivers, but also provides a better understanding of the older person’s habits. Such information may provide greater insight into the patient and the circumstances of the medical visit.

Potential of SHHTs in supporting older persons with medication management and self-care were evident in our findings. The technologies thus could improve the independence and well-being of older persons [142–144]. On a larger scale of population aging and the increase in older persons living with chronic diseases, this independence and reduced caregiving supervision also parallels with a call for self-management of one’s own health at home while adapting to “social, physical, and emotional challenges” [145]. Nonetheless, the individual differences to prefer independence or self-manage conditions, rather than to receive care from another family or professional caregiver, should also be taken into consideration. Peek and colleagues [146] presented these differences between older participants’ reactions to use computers or mobile phones for ordering groceries. Whereas some self-identified as being “stubborn, proud, [and] handle a lot of things by [them]selves” and would not want to rely on any assistance from caregivers or technology, other older participants selectively relied on certain types of technologies or simply on their children to complete these tasks digitally [146]. In the context of SHHTs, we also found similar differences that drove participants to prefer reduced human intrusion into the home and the use of reminder technologies for taking medications, while others preferred shared decision-making and in-person visits for these tasks [47, 78, 105, 130]. Whether an older person prefers to and would have greater confidence from independently accessing health information via technology, or would have greater trust in healthcare providers through in-person visits should also be considered.

Despite the potentials of SHHTs, it is more significant to highlight the several barriers which stand to hinder them from becoming viable solution for providing care for the older population [11, 147]. The most cited barrier was usability, which included issues such as difficult to use, obtrusive, intrusive, low in interoperability with existing household automation systems, and also technical limitations that needed to be overcome by developers themselves. As underlined by other studies, [147, 148], our systematic review further highlights that end-users had challenges in understanding the technology to the extent of avoidance and in particular, formal caregivers worried that the introduction of technologies could affect their work by requiring training and time for familiarization before allowing the older persons to use it independently. These practical challenges require critical thought and solutions from the part of technology developers. Improvement to user-friendliness could also improve access to the technologies and prevent the exacerbation of the digital divide. The articles reviewed revealed a wide range of knowledge and comfort levels of the designated end-users of SHHTs, where many of whom would likely be deterred by a technology that required lengthy training, frequent troubleshooting, and constant supervision. Conversely, a technology easily accessible and adoptable by more users could improve inequities amongst users of all backgrounds and technical expertise.

Though new developments of SHHTs were able to overcome and realize functional capabilities previously unseen, there were many technical malfunctions that caused frustration in end-users. Namely, there were challenges with the battery life, unstable or low wireless connectivity, incompatibility with existing house automation technologies, or that the technology was spatially inconvenient and could not fit comfortably in the home. Although these barriers seem simple and easy-to-overcome, they could be significantly troublesome for end-users to realistically implement in their home environment where familiarity is greatly valued. On the aspect of an incompatibility with existing home, there is call for adequate consideration towards the integration with existing home layouts while minimizing any modifications and interference to the older person [149]. As the aging process is dynamic and requires continuous adaptations from both caregivers and older persons, it is important that SHHTs are designed to interoperate with the end-users’ existing routines and home environment. Whilst the ability to access healthcare and constant remote monitoring could allow for a greater participation of the population living at a distance to healthcare resources or their caregivers, the extensive need for internet or cellular connection could again exclude older persons who are living in rural areas. This also plays into the need to incorporate large, bulky devices in the homes of older persons, begging the question of the characteristic or users whose home could meet these spatial requirements [150].

The aging process involves life transitions with higher risks of stressors, such as grief, bereavement, and a drop in socioeconomic statuses, which may in turn lead to an increased likelihood of loneliness and isolation for older adults [151, 152]. The technologies with a social function could allow older people to engage positively in group dynamics, reminiscence, or develop new ways to communicate with others. Confirming the findings from Latikka et al. [16], we discovered that SHHTs’ overall contribution to the reduction of loneliness was positive, albeit without complete elimination. Our search also contributed a nuanced perspective, whereby end-users felt that while SHHTs could improve the relationships and communication between caregivers and older persons, both in terms of quality and quantity, older persons still worried that these new functions may eventually replace the social connectedness that they enjoyed during in-person visits. To complement this ambivalence, it is helpful to look towards an alternative view (Zhu et al., 2021), where researchers recommended that technologies should act as a collaborator in human caregiving, instead of a substitute for care from children. The question whether technologies would or could or should replace human care is one that has been raised intensively [153–156], and necessitates critical and clear discussion among various stakeholders as to what the purpose of their technology is and how it fits the overall societal goals.

The dynamic and complex interactions of cultural values in the adoption of smart technologies may also extend the conversation beyond the technologies themselves. Though there are many mentions of a reduction in caregiving burden, it is interesting to examine the nuance in this argument from a cultural angle towards the presence of a smaller portion of articles with concerns for an increase in workload with the use of technology in caregiving. One study investigating the attitudes of low-income U.S. immigrant older persons towards remote monitoring systems cited high discontinuation of Korean and Chinese American residents, for fear of frequent false alerts or the increase of workload for their children [132]. Albeit also cited by other studies, a lack of cultural awareness and significant language barriers combined with false alerts from a passive monitoring system could leave “unforgettable negative experience[s]”, where an older user is found be left with embarrassment after a door is broken down by the emergency medical services. Although previous reviews have noted these already [18, 157], it is unfortunate that these recommendations have not been picked up and there still requires more development to improve the obtrusiveness and reliability of monitoring systems to reduce high rates of false alerts and malfunctions. Reducing feelings of embarrassment and maybe even stigma that follows are values that require serious attention.

Cost concerns has been raised by previous systematic reviews as a major barrier to adopting SHHTs [11]. While a stand-alone unit or an individual sensor may cost very little, to enable and maintain the continuous functioning of a complete and pervasive smart home environment could be more complicated than expected [5, 147]. Also included in this cost is the hiring of specialized individuals at a service center or in the hospitals, who would also need to invest more time and cost to understand, analyze, and effectively make use of the health data collected. In an article on the “Hidden Work” of implementing GPS tracking devices with emergency contact functions for persons with cognitive impairment, the authors report the human resources necessary for the continuous maintenance of a sustainable program, which include the coordinators, commissioners, occupational therapists, call operators and managers in monitoring centers to receive the calls, and technology suppliers [158]. On a larger scale, to implement the use of monitoring technologies on a system level requires greater costs for training staff, and interventions to shift habits towards using these new care tools [159]. Justice issues also come into play with the levels of accessibility of different older persons and their families, depending on their abilities to shoulder one-off and maintenance costs, comfort and knowledge in using technical tools for caregiving, and the friendliness of the local policies towards introducing SHHTs in their populations. These all influence and could exacerbate the digital divide that technologies are already bringing forth now.

The COVID-19 pandemic has forced many older persons around the world into isolation and quarantine at least during early months, which expectedly may lead to a different perspective and necessity to employ the technologies reviewed in this article [15, 160, 161]. Specifically, with the ability for remote monitoring, technologies could allow continuation of care across physical barriers and detection of rapid health declines, countering the effects of social isolation and feelings of reassurance. Due to the nature of reviewing empirical articles published on end-user feedback, those articles that were empirical and contextualized on COVID-19 concentrated on delivering telehealth services, reasonably answering to the demands of providing healthcare remotely, rather than elaborate SHHTs studied in this review. Nonetheless, despite not yet gathering empirical feedback from end-users, an abundance of smart technologies for caregiving of older persons has been developed during COVID-19 that are worthy of mention and shows promise for expediting the diversity of applicable scenarios for a diverse set of users [162, 163].

### Customization and policy-implications

Whilst customization was a theme that arose in the early studies, the ability for a more personalized approach to the design and implementation of SHHT systems that, if not increased in frequency, but at least persisted in recently published studies in greater detail [43, 53, 60, 86]. Users expressed that a heightened ability for technologies to “learn” and adapt its configurations, methods of data visualization, frequency of alerts, and complexity in its training programs could satisfy a more diverse array of user needs. This may be due to the improved technical capabilities and the shift towards personalized medicine in recent years. As a way to resolve individual preferences, comfort levels, or cultural norms, studies have cited the benefits of having technologies that were able to learn and adapt to the user [48]. For varying cost-bearing preferences and to accommodate the different national reimbursement programs, technologies should be offered in incremental stages with greater freedom for trial-periods before purchase. Directions of policy would greatly benefit the adoption or reduction of their barriers if it addresses the individual needs of older persons that are person-centric and situation-specific. Albeit smart homes are gradually making their way into policy decisions, there is a risk and uncertainty in their implementation to households that are of older persons or in rural areas with unstable wireless connectivity [164]. This would also heed calls for more personalization and tolerance in policy to adhere to the current array of needs and preferences.

### Limitations

This systematic review has limitations. Firstly, despite having searched in 10 electronic databases with a 22-year time-period limit to reasonably incorporate the span of technological development of SHHTs, some papers could have still been excluded from our search, for example, those archived in databases outside the ones we included. We stopped our search in December 2021, and thus were not able to include any publications from January 2022. In addition, as we opted to include only peer-review empirical articles, there could have been valuable insight in other forms of data output relevant to our topic that were published as, for example, theoretical papers, book chapters, thesis or have been published as grey literature. Related to this limitation is also the fact that since our work includes end users who were able to participate in studies. In lieu of existing digital divide, it also means that our review was unable to capture the view of those who could not access the technologies or were not interested to participate in these studies. Due to resource limitations (i.e. personnel time, limited funding period) as well as the high number of papers that were included in the full text evaluation, it was not possible to carry out independent double assessment of (a) each included paper as well as (b) complete the risk of bias of included studies. From the positive outcome that we reached by randomly checking 10% of the data for risks of content biases, as well as 10% of articles whose quality we assessed, we are cautiously confident about the quality of the included studies and the data extracted.

## Conclusions

Through functions of continuous monitoring, generating health-related reminders, providing additional channels for communication, SHHTs to-date could support caregivers in ways such as detecting falls and declines in functioning, provide assistance for basic activities for daily living, and the promotion and maintenance of a healthier lifestyle. However, our review found critical barriers to uptake that include issues with obtrusiveness and usability (such as technical problems and their limitations), social acceptance, costs, and the concern for loss of relationships. It becomes prudent to find ways to address these barriers as we move forward with technological development to ensure that the benefits generated does not come at higher costs. The data produced in this user-centric attempt to organize the current knowledge on SHHTs will prove informative to inform policy, improve user-acceptance, and serve as an additional resource for those who care for older persons. Finally, to take the call from Marikyan et al. [13] for further forwarding user-centric research, we encourage future researchers to focus on eliciting end-users’ conditions for acceptability in regards to different SHHTs. It would be interesting to also add independent variables in the equation, such as cultural background, generational gaps, technology readiness, living situation, financial comfort, and the nature of their social environment.

### Electronic supplementary material

Below is the link to the electronic supplementary material.

Supplementary Material 1

## Data Availability

The datasets used and/or analysed during the current study are available from the corresponding author on reasonable request. A supplementary table of the basic information from all articles analyzed for this review and an example of the search string is nevertheless included.

## Abbreviations

ADLs: Activities of daily living
IADLs: Instrumental activities of daily living
SHHTs: Smart home health technologies
IoTs: Internet of Things
