# Supplementary material for: Benefits and barriers associated with the use of smart home health technologies in the care of older persons: a systematic review
Source: BMC Geriatr. 2024 Feb 14;24:152. doi: 10.1186/s12877-024-04702-1 (PMC10865618; doi:10.1186/s12877-024-04702-1)
Supplement: Supplementary file 1 — Supplementary Material 1 [file 12877_2024_4702_MOESM1_ESM.docx]

**Appendices:**

§ **Complete Search String Example: EMBASE**

Population 1 + Intervention 1 + Population 2 + Context 1:

('aged'/exp OR 'aging'/exp OR 'geriatrics'/exp OR 'gerontology'/exp OR 'older adult':ti,ab OR 'ag*ng':ti,ab OR 'elder':ti,ab OR 'senior':ti,ab OR 'geriatr*':ti,ab OR 'gerontolo':ti,ab OR 'aged person':ti,ab OR 'old*':ti,ab) AND ('wireless communication'/exp OR 'assistive technology'/exp OR 'telemedicine'/exp OR 'gerontechnology'/exp OR 'smart home'/exp OR 'sensor'/exp OR 'wearable sensor'/exp OR 'wearable computer'/exp OR 'wearable device'/exp OR 'robotics'/de OR 'artificial intelligence'/exp OR 'information technology device'/exp OR 'information technology'/exp OR 'ambient intelligence'/exp OR 'welfare technology':ti,ab OR 'smart home':ti,ab OR 'smart home technolog*':ti,ab OR 'smart living':ti,ab OR 'home automation':ti,ab OR 'wireless home automation system*':ti,ab OR 'intelligent living':ti,ab OR 'intelligent building':ti,ab OR 'domotic*':ti,ab OR 'assistive domotic*':ti,ab OR 'embedded health system*':ti,ab OR 'ehealth':ti,ab OR 'health monitoring':ti,ab OR 'home-based health technology':ti,ab OR 'gerontechnology':ti,ab OR 'gerotechnology':ti,ab OR 'sensors':ti,ab OR 'wearable*':ti,ab OR 'robotic':ti,ab OR 'artificial intelligence in eldercare':ti,ab OR 'digital monitor*':ti,ab OR 'smart technologies to support healthy aging':ti,ab OR 'home-based assistive technolog*':ti,ab OR 'ambient assistive living':ti,ab OR 'intelligent assistive technolog*':ti,ab OR 'intelligent assistive device*':ti,ab OR 'intelligent assistive application':ti,ab) AND ('caregiver'/exp OR 'informal caregiver'/exp OR 'informal caregiving'/exp OR 'family caregiving'/exp OR 'care'/exp OR 'care and caring'/de OR 'nurse'/exp OR 'nursing'/exp OR 'nurse assistant'/exp OR 'physician'/de OR 'spouse'/exp OR 'adult child'/exp OR 'daughter'/exp OR 'wife'/exp OR 'husband'/exp OR 'son'/exp OR 'relative'/exp OR 'caregiv*':ab,ti OR 'informal caregiv*':ab,ti OR 'unpaid caregiv*':ab,ti OR 'famil* caregiv*':ab,ti OR 'care*':ab,ti OR 'formal caregiv*':ab,ti OR 'professional caregiv*':ab,ti OR 'nurse*':ab,ti OR 'nurse-aid*':ab,ti OR 'physician*':ab,ti OR 'doctor*':ab,ti OR spouse*:ab,ti OR 'adult child*':ab,ti OR 'daughter*':ab,ti OR 'wife':ab,ti OR 'husband':ab,ti OR 'son':ab,ti OR 'relative':ab,ti) AND ('home care'/exp OR 'nursing home'/exp OR 'independent living'/exp OR 'home'/exp OR 'house'/exp OR 'household'/exp OR 'home for the aged'/exp OR 'assisted living facility'/exp OR 'home care':ab,ti OR 'nursing home*':ab,ti OR 'independent living':ab,ti OR 'home*':ab,ti OR 'house*':ab,ti OR 'homes for the aged':ab,ti OR 'assisted living facilit*':ab,ti OR 'retirement home*':ab,ti) AND [embase]/lim AND [2000-2020]/py

**Chart of All Included Articles (N=163)**

| Last Name First Author | Title | Year | Study design | Studied technology* | Type of technology** |
| --- | --- | --- | --- | --- | --- |
| Airola*** | Domestication of a Robotic Medication-Dispensing Service Among Older People in Finnish Lapland | 2020 | Qualitative | d) | 1) 4) |
| Alexander | Sensor systems for monitoring functional status in assisted living facility residents | 2008 | Experimental | a) | 8) |
| Alexander | Evolution of an Early Illness Warning System toMonitor Frail Elders in Independent Living | 2011 | Mixed methods° | a) | 8) |
| Alexander | Passive Sensor Technology Interface to Assess Elder Activity in Independent Living | 2011 | Experimental | a) | 8) |
| Alexander | Generating Sensor Data Summaries to Communicate Change in Elder's Health Status | 2014 | Experimental | a) | 8) |
| Aloulou | Deployment of assistive living technology in a nursing home environment: methods and lessons learned | 2013 | Mixed methods | a) b) c) d) | 8) |
| Anderson*** | The Impact of Assistive Technologies on Formal and Informal Home Care | 2015 | Quantitative | d) | 9) |
| Annica | Summative evaluation of a sensor-based cognitive assistive technology: Impact on quality of life and perceived utility | 2019 | Mixed methods | d) | 3) |
| Arthanat | Caregiver perspectives on a smart home-based socially assistive robot for individuals with Alzheimer’s disease and related dementia | 2020 | Qualitative | b) d) | 1) |
| Baisch | Emotionale Roboter im Pflegekontext Empirische Analyse des bisherigen Einsatzes und derWirkungen von Paro und Pleo | 2017 | Mixed methods | c) | 1) |
| Bakas | Satisfaction and Technology Evaluation of a Telehealth Robotic Program to Optimize Healthy Independent Living for Older Adults | 2018 | Mixed methods | a) c) | 1) |
| Bankole | BESI: Behavioral and Environmental Sensing and Intervention for Dementia Caregiver Empowerment—Phases 1 and 2 | 2020 | Mixed methods | a) | 8) |
| Banks | Animal-assisted therapy and loneliness in nursing homes: use of robotic versus living dogs | 2008 | Mixed methods | c) | 1) |
| Barnier | Building Automation, an Acceptable Solution to Dependence? Responses Through an Acceptability Survey About a Sensors Platform | 2018 | Qualitative | a) b) | 8) |
| Barrett*** | Evaluation of a Companion Robot for Individuals with Dementia - Quantitative findings of the MARIO Project in an Irish Residential Care Setting | 2019 | Quantitative | c) | 1) |
| Bayen | Evaluating the effectiveness of a memory aid system | 2013 | Experimental | d) | 7) 8) |
| Bedaf | What are the preferred characteristics of a service robot for the elderly? A multi-country focus group study with older adults and caregivers | 2019 | Qualitative | d) | 1) |
| Berridge*** | Sensor-Based Passive Remote Monitoring and Discordant Values:Qualitative Study of the Experiences of Low-Income ImmigrantElders in the United States | 2019 | Qualitative | b) | 8) |
| Bertera | A study of the receptivity to telecare technology in a community-based elderly minority population | 2007 | Quantitative | a) b) | 3) 4) 5) 8) |
| Bevilacqua | Designing acceptable robots for assisting older adults: A pilot study on the willingness to interact | 2021 | Mixed methods | b) c) | 1) |
| Bharucha | Ethical considerations in the conduct of electronic surveillance research | 2006 | Qualitative | a) b) | 8) |
| Birks | Robotic Seals as Therapeutic Tools in an Aged Care Facility: A Qualitative Study | 2016 | Qualitative | c) | 1) |
| Blinka | Developing a sensor-based mobile application for in-home frailty assessmnet | 2021 | Qualitative | a) b) c) d) | 3) |
| Boise | Willingness of older adults to share data and privacy concernsafter exposure to unobtrusive in-home monitoring | 2013 | Quantitative | a) | 8) |
| Boissy | A qualitative study of in-home robotic telepresence for home care of community-living elderly subjects | 2007 | Qualitative | a) | 1) |
| Bowes | Telecare for Older People: Promoting Independence, Participation, and Identity | 2013 | Qualitative | b) | 8) |
| Bradford | Watching over me: positive, negativeand neutral perceptions of in-homemonitoring held by independent-livingolder residents in an Australian pilot study | 2018 | Qualitative | a) | 8) |
| Cahill | IoT/Sensor-Based Infrastructures Promoting a Senseof Home, Independent Living, Comfort and Wellness | 2019 | Qualitative | a) b) | 8) |
| Cai | Health professionals' user experience of the intelligent bed in patients' homes | 2015 | Mixed methods | a) | 8) |
| Canally | Using integrated bio-physiotherapy informatics in home health-care settings: A qualitative analysis of a point-of-care decision support system | 2015 | Mixed methods | a) | 8) |
| Cavallo | An ambient assisted living approach in designing domiciliary services combined with innovative technologies for patients with Alzheimer's disease: a case study | 2015 | Mixed methods | a) b) d) | 3) 4) 8) |
| Chaumon*** | Detecting falls at home: User-centered design of a pervasive technology | 2016 | Mixed methods | a) b) | 8) |
| Chung | Feasibility testing of a home-based sensor system to monitor mobility and daily activities in Korean American older adults | 2017 | Mixed methods | a) | 8) |
| Cohen | Acceptability of an intelligent wireless sensor system for the rapid detection of health issues: findings among home-dwelling older adults and their informal caregivers | 2016 | Mixed methods | a) | 8) |
| Corbett | Virtual assistant use and perceptions of usefulness by older adults and support person dyads | 2021 | Mixed methods | c) d) | 3) |
| Coşar | ENRICHME: Perception and Interaction of an Assistive Robot for the Elderly at Home | 2020 | Mixed methods | a) c) d) | 1) |
| De la Puente | Grasping Objects From the Floor in Assistive Robotics: Real World Implications and Lessons Learned | 2019 | Experimental | d) | 1) |
| Delmastro | Experimenting mobile and e-health services with frail MCI older people | 2019 | Experimental | a) | 8) |
| Dermody*** | Factors influencing community-dwelling older adults’ readiness | 2021 | Qualitative | a) b) | 8) |
| Draper | Ethical Values and Social Care Robots for Older People: An International Qualitative Study | 2017 | Qualitative | a) b) c) d) | 1) |
| Dupuy | Everyday functioning benefits from an assisted living platform amongst frail older adults and their caregivers | 2017 | Experimental | a) b) c) d) | 8) |
| Dupuy*** | Effects of an assisted living platform amongst frail older adults and their caregivers: 6 months vs. 9 months follow-up across a pilot field study | 2020 | Experimental | a) b) c) d) | 8) |
| Duque | Automation, Wellbeing and Digital Voice Assistants: Older People and Google Devices | 2021 | Qualitative | c) d) | 3) |
| Easton-Garrett | Utilizing artificial intelligence for falls management in memory care | 2020 | Experimental | b) | 8) |
| Eldib | Behavior analysis for elderly care using a network of low-resolution visual sensors | 2016 | Experimental | a) | 8) |
| Epstein*** | Older Adults' and Caregivers' Perspectives on In-Home Monitoring Technology | 2016 | Mixed methods | a) b) | 8) |
| Erebak | Caregivers’ Attitudes Toward Potential Robot Coworkers in Elder Care | 2019 | Experimental | a) c) | 1) |
| Essén | The two facets of electronic care surveillance: An exploration of the views of older people who live with monitoring devices | 2008 | Qualitative | a) b) | 3) |
| Faucounau | Caregivers' requirements for in-home robotic agent for supporting community-living elderly subjects with cognitive impairment | 2009 | Quantitative | a) b) c) d) | 1) |
| Fiorini | Assistive robots to improve the independent living of older persons: results from a needs study | 2021 | Qualitative | c) d) | 1) |
| Frennert | Elderly People's Perceptions of a Telehealthcare System: Relative Advantage, Compatibility, Complexity and Observability | 2013 | Qualitative | a) b) c) d) | 1) 8) |
| Gaiduk | Digital Health and Care Study on Elderly Monitoring | 2021 | Mixed methods | a) | 3) 8) |
| Geier | How nurses assess telepresence systems in outpatient care. A qualitative study | 2020 | Qualitative | c) | 1) |
| Ghorayeb | Older adult’s perspectives of smart home technology: Are we developing the technology that older people want? | 2021 | Qualitative | a) b) | 8) |
| Gokalp | Integrated Telehealth and Telecare for Monitoring Frail Elderly with Chronic Disease | 2018 | Experimental | a) b) | 8) |
| Görer | An autonomous robotic exercise tutor for elderly people | 2017 | Mixed methods | c) d) | 1) |
| Govercin | Defining the user requirements for wearable and optical fall prediction and fall detection devices for home use | 2010 | Mixed methods | b) | 3) 8) |
| Graf | Care-o-bot II - Development of a next generation robotic home assistant | 2004 | Quantitative | a) c) | 1) |
| Gullslett | Next of kin’s experiences with and attitudes towards digital monitoring technology for ageing people with dementia in residential care facilities. A qualitative study based on the voices of next of kin and care providers | 2021 | Qualitative | a) b) | 8) |
| Hall | Implementing monitoring technologies in care homes for people with dementia: A qualitative exploration using Normalization Process Theory | 2017 | Qualitative | a) b) d) | 8) |
| Holthe | A critical occupational perspective on user engagement of older adults in an assisted living facility in technology research over three years | 2020 | Mixed methods | a) b) | 8) |
| Hossain | Virtual Caregiver: An Ambient-Aware Elderly Monitoring System | 2012 | Mixed methods | b) | 8) |
| Huisman | Two-Year Use of Care Robot Zora in Dutch Nursing Homes: An Evaluation Study dagger | 2019 | Mixed methods | c) | 1) |
| Hunter | Issues associated with the management and governance of sensor data and information to assist aging in place: Focus group study with health care professionals | 2020 | Qualitative | a) b) | 8) |
| Hunter | Telehealth at Home: Co-Designing a Smart Home Telehealth System | 2021 | Qualitative | a) b) c) d) | 8) |
| Ide | The ageing ‘care crisis’ in japan: Is there a role for robotics-based solutions? | 2021 | Quantitative | c) | 1) |
| Iio | Social acceptance by senior citizens and caregivers of a fall detection system using range sensors in a nursing home | 2016 | Qualitative | b) | 8) |
| Inoue | Exploring the applicability of the robotic seal PARO to support caring for older persons with dementia within the home context | 2021 | Mixed methods | c) | 1) |
| Jenkins | Care, Monitoring, and Companionship: Views on Care Robots from Older People and Their Carers | 2015 | Qualitative | a) b) c) d) | 1) |
| Jo | Elderly perception on the internet of things-based integrated smart-home system | 2021 | Qualitative | a) b) | 3) 8) |
| Jøranson | Effects on sleep from group activity with a robotic seal for nursing home residents with dementia: a cluster randomized controlled trial | 2020 | Experimental | c) | 1) |
| Jøranson | Change in quality of life in older people with dementia participating in Paro-activity: a cluster-randomized controlled trial | 2016 | Experimental | c) | 1) |
| Junnila | Wireless, Multipurpose In-Home Health Monitoring Platform: Two Case Trials | 2010 | Experimental | a) b) | 8) |
| Jung | An Exploration of the Benefits of an animallike Robot Companion with more advanced touch interaction capabilities for Dementia Care | 2017 | Quantitative | c) | 1) |
| Khosla | Engagement and experience of older people with socially assistive robots in home care | 2021 | Mixed methods | c) d) | 1) |
| Kim | Unobtrusive Monitoring to Detect Depression for Elderly With Chronic Illnesses | 2017 | Mixed methods | a) | 8) |
| Kinney | Striving to Provide Safety Assistance for Families of Elders:The SAFE House Project | 2004 | Qualitative | b) | 8) |
| Klein | A robotic shower system: Acceptance and ethical issues | 2018 | Qualitative | b) d) | 1) |
| Kleiven | Health professionals' experiences with the implementation of a digital medication dispenser in home care services- A qualitative study | 2020 | Qualitative | a) b) | 4) |
| Klemets | Nurses' Perspectives on In-Home Monitoring of Elderlies's Motion Pattern | 2017 | Qualitative | a) b) | 8) |
| Korchut | Challenges for service robots-requirements of elderly adults with cognitive impairments | 2017 | Mixed methods | a) b) c) | 1) |
| Krakovski | "Gymmy": Designing and testing a robot for physical and cognitive training of older adults | 2021 | Quantitative | c) | 1) |
| Kramer | Comparison of the Effect of Human Interaction, Animal-Assisted Therapy, and AIBO-Assisted Therapy on Long-Term Care Residents with Dementia | 2009 | Mixed methods | c) | 1) |
| Lamprinakos | An integrated remote monitoring platform towards Telehealth and Telecare services interoperability | 2015 | Quantitative | a) b) | 8) |
| Law | Developing assistive robots for people with mild cognitive impairment and mild dementia: A qualitative study with older adults and experts in aged care | 2019 | Qualitative | a) b) c) d) | 1) |
| Lazarou | A Novel and Intelligent Home Monitoring System for Care Support of Elders with Cognitive Impairment | 2016 | Mixed methods | a) b) d) | 8) |
| Lee | Sensor-based observations of daily living for aging in place | 2015 | Qualitative | a) | 8) |
| Lee | Toward a Sociable and Dependable Elderly Care Robot: Design, Implementation and User Study | 2020 | Mixed methods | c) d) | 1) |
| Liang | A Pilot Randomized Trial of a Companion Robot for People With Dementia Living in the Community | 2017 | Experimental | c) | 1) |
| Libin | Therapeutic robocat for nursing home residents with dementia: Preliminary inquiry | 2004 | Experimental | c) | 1) |
| Londei | An intelligent videomonitoring system for fall detection at home: perceptions of elderly people | 2009 | Mixed methods | b) | 8) |
| Lussier | Integrating an Ambient Assisted Living monitoring system into clinical decision-making in home care: An embedded case study | 2020 | Mixed methods | a) b) | 8) |
| Mahoney | An evidence-based adoption of technology model for remote monitoring of elders' daily activities | 2011 | Mixed methods | a) b) | 8) |
| Marti*** | Exploring empathy in interaction | 2013 | Qualitative | a) c) d) | 1) |
| Matsui | Development of a practicable non-contact bedside autonomic activation monitoring system using microwave radars and its clinical application in elderly people | 2013 | Experimental | a) | 8) |
| Mazzu | Wireless-accessible sensor populations for monitoring biological variables | 2008 | Quantitative | a) | 8) |
| Meiland | Participation of end users in the design of assistive technology for people with mild to severe cognitive problems; the European Rosetta project | 2014 | Qualitative | a) b) | 8) |
| Melkas | Innovative assistive technology in Finnish public elderly-care services: A focus on productivity | 2013 | Mixed methods | a) b) c) | 3) 4) 5) 8) |
| Melkas | Impacts of robot implementation on care personnel and clients in elderly-care institutions | 2020 | Mixed methods | c) d) | 1) |
| Milligan | Telecare and older people: Who cares where? | 2011 | Qualitative | a) b) c) d) | 4) 8) |
| Mitseva | Gerontechnology: Providing a helping hand when caring for cognitively impaired older adults-intermediate results from a controlled study on the satisfaction and acceptance of informal caregivers | 2012 | Experimental | a) b) c) d) | 8) |
| Morris | Social networks as health feedback displays | 2005 | Qualitative | c) | 3) |
| Moyle | Use of a Robotic Seal as a Therapeutic Tool to Improve Dementia Symptoms: A Cluster-Randomized Controlled Trial | 2017 | Experimental | c) | 1) |
| Mucchiani | Exploring Low-Cost Mobile Manipulation for Elder Care Within a Community Based Setting | 2020 | Mixed methods | b) c) d) | 1) |
| Naick | Innovative approaches of using assistive technology to support carers to care for people with night-time incontinence issues | 2017 | Case study | a) | 8) |
| Niemelä | Towards Ethical Guidelines of Using Telepresence Robots in Residential Care | 2019 | Mixed methods | b) c) | 1) |
| Npochinto | Intérêt en EHPAD du robot émotionnel  Pepper dans les troubles  neurocomportementaux de la maladie  d’Alzheimer | 2021 | Experimental | c) | 1) |
| O'Brien | Voice‐Controlled Intelligent Personal Assistants to Support Aging in Place | 2020 | Qualitative | c) | 6) |
| Obayashi | Can connected technologies improve sleep quality and safety of older adults and care-givers? An evaluation study of sleep monitors and communicative robots at a residential care home in Japan | 2020 | Mixed methods | a) b) | 8) |
| Obayashi | Pilot and Feasibility Study on Elderly Support Services Using Communicative Robots and Monitoring Sensors Integrated With Cloud Robotics | 2020 | Mixed methods | a) b) | 1) 8) |
| Ohta | A health monitoring system for elderly people living alone | 2002 | Observational | b) | 8) |
| Pais*** | Evaluation of 1-Year in-Home Monitoring Technology by Home-Dwelling Older Adults, Family Caregivers, and Nurses | 2020 | Mixed methods | a) b) | 8) |
| Patel*** | A passive monitoring system in assisted living facilities: 12-month comparative study | 2012 | Experimental | b) | 8) |
| Pazhoumand-Dar | Detecting deviations from activities of daily living routines using kinect depth maps and power consumption data | 2020 | Quantitative | b) | 8) |
| Pérez | Caregiver and social assistant robot for rehabilitation and coaching for the elderly | 2015 | Quantitative | c) d) | 1) |
| Peter | AGNES: Connecting people in a multimodal way | 2013 | Mixed methods | a) c) | 8) |
| Pfadenhauer | Robot Caregiver or Robot-Supported Caregiving?: The Performative Deployment of the Social Robot PARO in Dementia Care | 2015 | Qualitative | c) | 1) |
| Pineau | Towards robotic assistants in nursing homes: Challenges and results | 2003 | Experimental | c) d) | 1) |
| Pol | Effectiveness of sensor monitoring in a rehabilitation programme for older patients after hip fracture: A three-arm stepped wedge randomised trial | 2019 | Experimental | a) b) | 8) |
| Pu | The Effect of Using PARO for People Living With Dementia and Chronic Pain: A Pilot Randomized Controlled Trial | 2020 | Experimental | c) | 1) |
| Rantanen*** | An In-home Advanced Robotic System to Manage Elderly Home-care Patients’ Medications: A Pilot Safety and Usability Study | 2017 | Mixed methods | d) | 4) |
| Rantz | Randomized Trial of Intelligent Sensor System for Early Illness Alerts in Senior Housing | 2017 | Experimental | a) | 8) |
| Rawtaer | Early detection of mild cognitive impairment with in-home sensors to monitor behavior patterns in community-dwelling senior citizens in Singapore: Cross-sectional feasibility study | 2020 | Experimental | a) b) | 4) 8) |
| Reeves | A trial of telecare for supporting care to the elderly in Liverpool | 2007 | Experimental | a) b) | 8) |
| Robert | SWEET-HOME ICT technologies for the assessment of elderly subjects | 2013 | Quantitative | a) b) d) | 8) |
| Robinson*** | Technology for healthy independent living: Creating a tailored in-home sensor system for older adults and family caregivers | 2020 | Qualitative | a) | 8) |
| Ropero | LARES: An AI-based teleassistance system for emergency home monitoring | 2019 | Case study | a) b) c) | 1) 8) |
| Rose | Correlates Among Nocturnal Agitation, Sleep, and Urinary Incontinence in Dementia | 2015 | Mixed methods | a) | 8) |
| Rostill | Technology-integrated dementia care: trial results | 2019 | Mixed methods | a) b) | 8) |
| Rowe*** | Sleep in Dementia Caregivers and the Effect of a Nighttime Monitoring System | 2010 | Mixed methods | a) b) | 8) |
| Sadek | Nonintrusive Remote Monitoring of Sleep in Home-Based Situation | 2018 | Observational | a) | 8) |
| Salichs | Mini: A New Social Robot for the Elderly | 2020 | Quantitative | c) | 1) |
| Sanchez | Older people’s attitudes and perspectives of welfare technology in Norway | 2019 | Qualitative | a) b) | 8) |
| Sanchez | ICT Services for Life Improvement for the Elderly | 2017 | Iterative Testing | a), b) c) | 8) |
| Saunders | 'Teach Me-Show Me'-End-User Personalization of a Smart Home and Companion Robot | 2016 | Mixed methods | c) | 1) 8) |
| Seelye | Reactions to a remote-controlled video-communication robot in seniors' homes: A pilot study of feasibility and acceptance | 2012 | Experimental | c) d) | 1) |
| Sixsmith*** | An evaluation of an intelligent home monitoring system | 2000 | Mixed methods | b | 8) |
| Snyder | Remote monitoring technologies in dementia care: An interpretative phenomenological analysis of family caregivers’ experiences | 2020 | Qualitative | a) b) c) | 3) 6) 8) |
| Sung | Robot-assisted therapy for improving social interactions and activity participation among institutionalized older adults: A pilot study | 2015 | Experimental | c) | 1) |
| Suryadevara | Wireless Sensor Network Based Home Monitoring System for Wellness Determination of Elderly | 2012 | Experimental | b) | 8) |
| Suwa | Exploring perceptions toward home-care robots for older people in Finland, Ireland, and Japan: A comparative questionnaire study | 2020 | Quantitative | a) b) c) d) | 1) |
| Tamura | Is an Entertainment Robot Useful in the Care of Elderly People with Severe Dementia? | 2004 | Experimental | c) | 1) |
| Tanaka | Effect of a human-type communication robot on cognitive function in elderly women living alone | 2012 | Experimental | d) | 1) |
| Tang | An IoMT-based geriatric care management system for achieving smart health in nursing homes | 2019 | Experimental | a) | 3) |
| Thilo | Facilitating the use of personal safety alerting device with older adults: The views, experiences and roles of relatives and healthcare professionals | 2021 | Qualitative | a) b) | 8) |
| Tiersen | Smart home sensing and monitoring in households with dementia: User-centered design approach | 2021 | Mixed methods | a) b) c) d) | 8) |
| Torta | Evaluation of a Small Socially-Assistive Humanoid Robot in Intelligent Homes for the Care of the Elderly | 2014 | Experimental | c) | 1) |
| Tseng | Designing an intelligent health monitoring system and exploring user acceptance for the elderly | 2013 | Experimental | a) | 8) |
| Tulsukar | Can a humanoid social robot stimulate the interactivity of cognitively impaired elderly? A thorough tsudy based on computer vision methods | 2021 | Observational | c) | 1) |
| Urwyler | Cognitive impairment categorized in community-dwelling older adults with and without dementia using in-home sensors that recognise activities of daily living | 2017 | Observational | a) | 8) |
| Valenti | Social robots in advanced dementia | 2015 | Experimental | d) | 1) |
| Van Berlo | Experiences with smart homes for older people | 2011 | Observational | b) | 8) |
| Vandenberg | US and Dutch nurse experiences with fall prevention technology within nursing home environment and workflow: A qualitative study | 2017 | Qualitative | b) | 8) |
| VandeWeerd | HomeSense: Design of an ambient home health and wellness monitoring platform for older adults | 2020 | Experimental | a) | 8) |
| Verloo | Perceptions About Technologies That Help Community-Dwelling Older Adults Remain at Home: Qualitative Study | 2020 | Qualitative | a) b) d) | 9) |
| Wai | Smart wireless continence management system for persons with dementia | 2008 | Observational | a) | 9) |
| Wang | A Personalized Health Monitoring System for Community-Dwelling Elderly People in Hong Kong: Design, Implementation, and Evaluation Study | 2020 | Quantitative | a) b) | 3) 8) |
| Wang | Robots to assist daily activities: Views of older adults with Alzheimer's disease and their caregivers | 2017 | Qualitative | c) | 1) |
| Wilkinson | Monitoring Health Status in Long Term Care Through the Use of Ambient Technologies and Serious Games | 2018 | Experimental | a) | 8) |
| Woods | Subverting the logics of “smartness” in Singapore: Smart eldercare and parallel regimes of sustainability | 2020 | Qualitative | a) b) | 8) |
| Wright | Tactile care, mechanical Hugs: Japanese caregivers and robotic lifting devices | 2018 | Qualitative | b) | 1) |
| Yamakazi | Anxiety Reduction Through Close Communication with Robotic Media in Dementia Patients and Healthy Older Adults | 2020 | Experimental | c) d) | 1) |
| Zsiga*** | Home care robot for socially supporting the elderly: Focus group studies in three European countries to screen user attitudes and requirements | 2013 | Qualitative | a) b) c) | 1) |

*(a) physiological and functional monitoring; (b) safety/security monitoring and assistance; (c) social interactions; and (d) cognitive and sensory assistance

**(1) assistive autonomous robots; (2) self-driving vehicles; (3) AI-enabled health smart apps and wearables; (4) new drug release mechanisms; (5) portable diagnostics; (6) voice-activated devices; (7) virtual reality (VR), augmented reality (AR), and mixed reality (MR); and (8) intelligent homes (Abdi, de Witte & Hawley, 2020). (9) Other

*** Quality assessment of these studied were performed using appropriate Critical Appraisal Skills Program checklist (qualitative studies, case control, cohort etc.)

° Mixed methods were those studies that used (a) qualitative and quantitative data collection methods and/or (b) analyzed the collected data using both qualitative and quantitative methods.
